# International Investment and Indigenous Peoples’ Environment: A Survey of ISDS Cases from 2000 to 2020

**DOI:** 10.3390/ijerph18157798

**Published:** 2021-07-22

**Authors:** Chao Wang, Jing Ning, Xiaohan Zhang

**Affiliations:** 1 Academy of International Strategy and Law, Zhejiang University, Hangzhou 310008, China; superwang@zju.edu.cn; 2 Guanghua Law School, Zhejiang University, Hangzhou 310008, China; ningjing@zju.edu.cn

**Keywords:** indigenous peoples, environment, human rights, international investment law, investor-state dispute settlement, free, prior and informed consent

## Abstract

Indigenous peoples’ environments can be easily disrupted by foreign investments, and disputes have occasionally occurred over the past few years. The objective of this research article is to examine if current international investment law, especially its investor-state dispute settlement (ISDS) mechanism, could provide necessary protection to Indigenous rights. We searched all publicly available ISDS cases from 2000 to 2020, and selected 10 typical ones for comprehensive case study by using various research methods such as doctrinal legal research and comparative analysis. Our research revealed that Indigenous peoples’ participation in the ISDS proceedings is legally restrained, time-consuming, and rarely favorably decided by the arbitral tribunals. Responsibility for such undesirable outcomes rests with all stakeholders involved in the process, while the consequences of post-arbitration tend to be “triple losing”. These findings highlight the quest for a more sustainable international investment regime that promotes Indigenous peoples’ wellbeing and environment protection. We argue that future reform could be promoted not only over ISDS procedural matters, but also by upgrading substantive rules in international investment agreements (IIAs), emphasizing free, prior, and informed consent (FPIC), and strengthening foreign investors’ corporate social responsibilities (CSR).

## 1. Introduction

“Indigenous peoples” refers to “indigenous populations that live within, or attached to, geographically distinct traditional habitats or ancestral territories, and who identify themselves as being part of a distinct cultural group, descended from groups present in the area before modern states were created and current borders defined” [1]. The United Nations estimates that there are more than 370 million Indigenous people spread across 70 countries worldwide, such as the Lakota in the United States, the Mayas in Guatemala, the Saami of northern Europe, the Aboriginal and Torres Strait Islander peoples of Australia, and the Maori of New Zealand. [2]. Indigenous peoples’ rights, including their right to a healthy and sustainable environment, are widely acknowledged by the international community and protected by a number of international legal instruments. For example, the Convention (No. 169) concerning Indigenous and Tribal Peoples in Independent Countries, adopted by the International Labour Organization in June 1989, requires that national governments “shall take measures … to protect and preserve the environment of the territories they inhabit” [3]. The United Nations Declaration on the Rights of Indigenous Peoples (UNDRIP), which is by far “the most comprehensive international instrument on the rights of indigenous peoples” [4], emphasizes protection of their rights to lands and recourses, as well as the free, prior, and informed consent (FPIC) principle [5].

Indigenous peoples mostly reside in natural resource-rich regions that are more likely to attract international investment made by foreign investors, whether operating companies or a natural person. A 2015 World Bank study on the current global land rush showed that “higher commodity prices and concerns about food security, a history of under investment in agriculture, and wide variation in land scarcity and productivity across countries have considerably increased interest by investors in agricultural land” [6]. These land development activities, especially in the Global South, have a direct impact on local Indigenous peoples, some of whom “are often forced to either endure enclosure or move to more isolated, marginal locations” [7]. Consequently, there are frequent news reports and studies revealing that, apart from promoting local economic development and modernization of the host states, foreign investments might also result in environment damages [8]. Some disastrous consequences of the occupation of the traditional territories of Indigenous peoples include the destruction of their dwellings and deforestation, resulting in pollution or even cutting off of drinking water sources, causing tremendous suffering to local Indigenous communities and leading to tragic incidents and disputes. A recent example was reported in March 2020. Mass demonstrations, sit-ins, and blockades gripped parts of Canada as part of a movement to support the Indigenous Wet’suwet’en Nation’s hereditary chiefs, who were opposed to a 670-kilometre pipeline project that would cut across traditional Wet’suwet’en lands in northern British Columbia [9].

To address potential conflicts that can arise between foreign investors, Indigenous peoples, local governments of the host states, and international organizations have gradually developed certain policies and legal mechanisms in recent years. For instance, in 2015 the United Nations Conference on Trade and Development (UNCTAD) formulated an Investment Policy Framework for Sustainable Development (IPFSD) aiming to resolve issues stemming from the increasing complexity of the international investment policy regime [10]. Under the international law framework, rights and obligations of the host states and foreign investors are primarily regulated by international investment agreements (IIAs) such as bilateral investment treaties (BITs) and treaties with investment provisions (TIPs). According to UNCTAD statistics, by the end of 2020 there were 2875 BITs and 417 TIPs [11]. These legal instruments broadly cover issues of market access, standards of protection, compensation for losses, and limitations on requisition and expropriation. For disputes arising from investment activities, IIAs usually define procedures on investor-state dispute settlement (ISDS), giving foreign investors the right to submit a dispute to an international arbitral tribunal. Most of the ISDS cases are administered by well-established international institutions such as the International Centre for Settlement of Investment Disputes (ICSID) and the Permanent Court of Arbitration (PCA). In recent years, “intersections between public health regulation and trade and investment agreements have become more common” [12], and the ISDS mechanism is “often seen as a threat or limitation on the ability of states to enact measures for the protection of public health” [13].

There is an abundance of research in the fields of international investment law, sustainable development, public health, and environment protection. However, specific research focusing on their interaction, especially in relation to Indigenous peoples’ environmental rights, appears to be limited. Few scholars have invoked interdisciplinary concepts such as intergenerational environmental justice and Indigenous sovereignty [14]. Lorenzo Cotula’s latest article, for example, “holistically examines the intersections between developments often discussed in separate fields—from land law to investment treaties” [15]. The existing literature largely focuses on textual studies of the IIAs, i.e., interpretation of relevant legal provisions [16], or dynamics of complementarity between international law and individual national law [17], and we did not find any comprehensive and empirical study of relevant ISDS cases. As a consequence, some practical issues remain unaddressed: How many related disputes have been submitted to arbitration and what are the outcomes? How effective and efficient is the ISDS mechanism in terms of protecting Indigenous peoples’ environment? What are the implications and what reforms need to be introduced? Seeking answers to these questions is the main aim of this research.

To this end, we selected a number of typical ISDS cases that were adjudicated during the period from 2000 to 2020. After analyzing proceedings and outcomes of these cases, we looked for the underlying rationale. We discovered that Indigenous communities’ participation of ISDS proceedings is far from being active and easy, while arbitral tribunals inadequately consider Indigenous peoples’ environmental protection as an important factor in their adjudications of investor-state disputes. ISDS proceedings generally end in favor of foreign investors. As a consequence, Indigenous communities who are denied a satisfactory legal remedy would lose their traditional habitats, leading to violent protests against foreign investors in order to safeguard their territories and environment. After further analyzing the systemic deficiencies of the ISDS mechanism, we conclude that both substantive rules and procedures under the current international investment law regime need to be reformed to better protect Indigenous peoples’ environment and promote sustainable development.

## 2. Materials and Methods

### 2.1. Data and Materials Collection

For publicly available non-confidential ISDS cases involving Indigenous peoples, we primarily searched ITALAW, the most comprehensive database on international investment law and investor-state arbitration, by using the keyword “Indigenous” and setting the case registration period between year 2000 and 2020. We also searched official websites of major ISDS institutions such as the ICSID and the PCA, to check if there are additional cases that are relevant but not included by the ITALAW.

For secondary sources, i.e., interpretation of relevant treaty provisions of IIAs, the UNDRIP, and the IPFSD, as well as commentaries on arbitral awards rendered by ISDS tribunals, research literature, and references, we used Heinonline, Westlaw, LexisNexis, and other professional legal databases.

With respect to supplementary facts about Indigenous peoples and their environment, their conflicts with foreign investors, developments of policy-making and legislations on protecting their environmental rights, relevant information, and research materials, we mainly used official websites of involved institutions, such as the Office of the United Nations High Commissioner for Human Rights (OHCHR) which has a special section on Indigenous peoples, the United Nations Permanent Forum on Indigenous Issues (UNPFII), and other open access resources and international news reports.

### 2.2. Methods

This research is conducted primarily based on a comprehensive study of relevant ISDS cases. First, we identified the selection criteria for international investment disputes, including their relevance to environment protection and involvement of Indigenous peoples, within the time frame of 2000 to 2020. After making a selection of target cases, we carried out comprehensive cases studies. In particular, we concentrated on those representative cases that have significant implications for both foreign investors and Indigenous peoples. We carefully investigated the participation of Indigenous communities in ISDS proceedings, duration of the proceedings, decisions of the arbitral tribunals, and the post-arbitration results.

Other well-established methods such as doctrinal research methodology in legal research were also regularly adopted. We critically analyzed the texts of the relevant legal provisions in various IIAs, the UNDRIP, and international conventions. National laws of some individual countries such as the United States and Canada are also referred to. Through such “black letter law” analysis, we further identified some ambiguities, inefficiencies, and even inconsistencies in the existing ISDS system, based on which we propose improvement offering suggestions and solutions.

Comparative studies are used both on ISDS cases and on black-letter provisions. For instance, the Republic of Ecuador was sued by foreign investors in ICSID and by Indigenous communities in human rights court. By comparing the two different legal proceedings as well as the results of adjudication, we acquired a more comprehensive and deeper understanding of the ISDS mechanism. We also compared various legal texts such as provisions on third-parties’ participation as stipulated by the ICSID Convention, the UNCITRAL Arbitration Rules, and other IIAs.

## 3. Results

### 3.1. Cases and Indigenous Peoples involved

By using the above-mentioned criteria and methods, we searched all publicly available ISDS cases adjudicated between 2000 and 2020, and found that there are at least 20 cases involving, directly or indirectly, Indigenous peoples. We further studied the essentials of these cases, including the arbitrating authorities, applicable laws, parties and merits of disputes, and identified 10 typical ones that are in close connection with Indigenous peoples’ environmental rights (Table 1).

These ISDS cases reveal that, when Indigenous peoples are involved in foreign investment development activities, their land, water, natural resources, and environment are very likely to be adversely affected. In the past two decades, disputes over environmental protection constantly occurred between foreign investors and local Indigenous communities of the host state, broadly covering major Indigenous peoples residing in North America, Latin America, and Africa. In most cases, Indigenous peoples’ environmental rights were not infringed directly by foreign investors who were legally licensed to exploit by the local governments for the purpose of “developing local economy”. The applicable laws in these cases, mostly BITs, usually do not expressly provide for the protection of Indigenous peoples’ environment.

### 3.2. Arbitration Proceedings

With respect to the ISDS proceedings, we found that in nearly all cases, participation of Indigenous communities in the arbitration procedures was neither easy nor active (Table 2).

It is only in a few cases, such as the Glamis Gold, Ltd. v. United States of America and the Grand River Enterprises Six Nations, Ltd., et al., v. United States of America, that Indigenous communities or their representatives directly participated in the proceedings by means of submitted petitions or other documentation, and in the capacity of amicus curiae or non-disputing parties (NDPs). In most cases, their applications for participation were rejected by the ISDS tribunals. In comparison, some human rights or environmental NGOs, such as the Peruvian human rights organization in the Bear Creek Mining Corp. v. Republic of Peru case and the European Center for Constitutional and Human Rights (ECCHR) in the Bernhard von Pezold and others v. Zimbabwe case, were more active than the Indigenous communities in the participation of ISDS proceedings.

In addition, most of the ISDS proceedings lasted several years. For example, in the South American Silver Limited v. Bolivia case, the Notice of Arbitration was submitted in April 2013, but the final award was not rendered by the PCA tribunal until November 2018. Another typical case decided by the ICSID tribunal, the Pac Rim Cayman LLC v. Republic of El Salvador, took even a longer time: it was initiated in December 2008, but the final award was rendered in October 2016, and a Supplementary Decision was made in March 2017, nearly nine years later.

### 3.3. Arbitration Results

With respect to the arbitration results, no matter whether the final decision was in favor of foreign investors or the host state, we found that in most cases the ISDS tribunals did not take into account Indigenous peoples’ environment protection as an important factor in their adjudication of investor-state disputes (Table 3). Even if in several cases the tribunal accepted Indigenous communities’ application for participation, it rarely took their representation into account in the final award. In other words, even if the Indigenous communities successfully appeared in the ISDS proceedings, their petitions and submissions were rarely considered decisive for the case.

We further found that the consequences of post-arbitration are sometimes “triple losing”: as Indigenous peoples’ petitions were not satisfyingly addressed in the ISDS proceedings and their environmental interests therefore suffered damages, some Indigenous communities chose to continue their struggle through violent protests, attacking and destroying foreign investors’ factories and properties. Being unable to secure economic interests and even running at a loss, some foreign investors withdrew their investments, and later sued the host states for compensation for losses by claiming that the host states failed to fulfill their international obligations under IIAs. Host states, as the ultimate stakeholder, not only lost opportunities to develop local economy by taking advantage of foreign capital, but also had to bear the costs of ecological restoration.

## 4. Discussion

### 4.1. Causation

Conserving Indigenous peoples’ interests and wellbeing amidst international investment development activities has been a complicated challenge for many host states, as it involves multiple issues of economic and social policies, national laws, human rights, and the environment. As mentioned above, because of the natural tension among foreign investors, local governments, and Indigenous communities over economic development and environment protection, disputes have constantly occurred in the past two decades. Some underwent legal proceedings such as international arbitration. However, current international investment law, including its ISDS mechanism, can hardly provide necessary legal remedies for Indigenous peoples to conserve their environment. In terms of the causation for such a situation, we deem that all stakeholders involved in the ISDS system are concerned.

#### 4.1.1. Host States

Host states are not in a simply fixed position in face of foreign investors and Indigenous peoples. Their roles in protecting Indigenous rights are also different to those of the foreign investors. When economic development activities are environment-friendly, and Indigenous communities are in peaceful coexistence with foreign investors, host states tend to provide more favorable treatment to investors so as to increase revenue and promote employment. Nevertheless, if host states fail to fulfill their international obligations (e.g., duty to consult) prior to granting licences to foreign investors, or neglect their own responsibilities under international law, local Indigenous communities are consequently in serious conflict with foreign investors, and domestic pressure for local protection is high, host states are likely to adjust their position: they may revoke previous commitments, and even expropriate foreign investors’ investment and properties, by invoking ‘legitimate provisions’ such as public interests and environment protection. Though it is often the host states’ initial failure to fulfill their own obligations that results in conflict between Indigenous peoples and foreign investors, when disputes arise, as noted by the amicus curiae in the Pac Rim Cayman LLC v. Republic of EI Salvador case, “at most, the State acted only as an intermediary between the Claimant [foreign investors] and the communities” [18].

From the regulatory perspective, current IIAs usually do not provide regulations or guidance for host states in dealing with conflicts between foreign investors and Indigenous peoples, while a host state might be held accountable if protection of Indigenous peoples’ environment causes loss to the foreign investor. As such, the ISDS mechanism “has shifted from being a shield of last resort to a sword of first resort in many disputes” between host states and foreign investors [19]. Under such a legal environment, host states are in a risky position and therefore have even less incentive to protect Indigenous peoples and their environment.

#### 4.1.2. Foreign Investors

Usually, the priority of foreign investors is their economic interests, i.e., financial aspect [20], rather than conservation of Indigenous peoples’ environment. Current international investment laws too place emphasis more on promoting and protecting investments than upholding human rights or protecting the environment. In such a legal environment, it is understandable that profit-driven investors have poor motivation to actively consider Indigenous rights when making investment decisions. Even if they attend environmental public forums to interact with local residents, they are not obliged to follow up on those suggestions [21]. It is only when their investments are obstructed or severely impacted, for example by Indigenous peoples’ protests, that foreign investors might take environment into account, start negotiations with the affected Indigenous communities, and make adjustments to their development plan or compensation for environmental damages.

From the regulatory perspective, IIAs, especially BITs, are designed primarily to regulate states—home states of foreign investors and host states of the investments. Foreign investors are not legally bound by these international investment instruments which bind only state parties. Their legal liabilities, including compensation for environmental damages, are largely derived from violation of municipal laws of the host states. As a result, costs for breaking laws in this regard are generally not high. Furthermore, host states are reluctant to be involved into time-consuming proceedings against foreign investors. These factors further lead foreign investors to be much less concerned with of the need to protect Indigenous peoples and their environment.

#### 4.1.3. Indigenous Peoples

When facing foreign investments, Indigenous peoples’ situation may vary from one country to another. In countries such as China, even the “Indigenous communities are given more autonomy to govern their common resources” [22], different regions “are developing their own individual responses to environmental conservation” while being influenced by global norms [23]. But in general, Indigenous peoples normally lack competence in effectively participating in political and legal affairs. They have a weak voice and influence over domestic matters of granting licenses to foreign investors. Even when they have objections to the investment development plans, Indigenous communities are rarely consulted by the local governments, especially in developing and least developed countries. With respect to international judicial activities, such as highly professionalized ISDS proceedings which are time-consuming and prohibitively expensive, Indigenous peoples usually lack of the necessary capacity to participate. All these are genuine difficulties for Indigenous peoples in safeguarding their interests and environment.

Even if the affected Indigenous communities have the intention and capability to resort to ISDS proceedings, they are only entitled to participate as amici curiae or NDPs other than as claimants of the case. According to ICSID Arbitration Rule 32(2), where a party objects to the request of an NDP to attend the hearings in a proceeding, a tribunal has no discretion to grant such a request over that party’s objection. In other words, Indigenous communities have neither initiative power nor an independent position in taking advantage of the ISDS proceedings.

#### 4.1.4. ISDS Arbitrators

Arbitrators also play a key role. In the Bernhard von Pezold and others v. Zimbabwe case, four Indigenous communities, namely the Chikukwa, Ngorima, Chinyai, and Nyaruwa peoples, together with the ECCHR applied to participate as amicus curiae and submitted three petitions, but were denied in entirety. The tribunal held that it had the discretion, upon consulting with the Parties, to allow an NDP to file a written submission pursuant to ICSID Arbitration Rule 37(2), provided that certain minimum criteria were met [24]. In the Bear Creek case, one submission from an American think tank was rejected as the arbitrators were of the view that it could not “contribute any further information or arguments that would assist the tribunal” [25]. More similar cases also indicate that, apart from being subject to other factors, whether Indigenous peoples could resort to the ISDS mechanism rests with the arbitrators’ discretion.

Most ISDS arbitrators are experts of international investment law, but may not have strong backgrounds or awareness of human rights and environmental protection. Past ISDS practice shows that some ISDS arbitrators display more or less a distinct neglect or ignorance of the need for taking proper account of incorporating Indigenous peoples’ interests in investor-state dispute adjudication. Some vital international treaties on human rights, such as the International Covenant for Civil and Political Rights (ICCPR), the UN Convention for the Elimination of All Forms of Racial Discrimination (CERD), and the African Charter on Human and Peoples’ Rights, were not seriously treated or respected. Among the cases covered by this present study, we also did not find even one case in which the arbitrators expressly highlighted protection of Indigenous peoples’ environmental rights. In the Border Timbers case, the tribunal even maintained they are not persuaded that “consideration of the foregoing [protecting Indigenous peoples] is in fact part of their mandate under either the ICSID Convention or the applicable BITs” [26].

### 4.2. Solutions

As the findings of this study reveal, the current ISDS mechanism under international investment law framework can hardly provide full protection to Indigenous peoples’ environment. The International Law Association Committee noted that, concerning the applicable laws with regard to Indigenous peoples’ rights, the protection stems from interplay between the domestic, national, regional, and international levels [27]. To strengthen protection and promote sustainable development, we propose reform in the following areas:

#### 4.2.1. ISDS Procedural Matters

Procedural matters of the ISDS mechanism are apparent and relatively easy to address. One outstanding issue, as this study reveals, is that Indigenous peoples can participate only as amici curiae or NDPs other than as disputing parties, and whether their petitions could be taken into account is largely subject to the tribunals’ discretion. Arbitrary decisions to ignore Indigenous rights were frequently seen in past ISDS proceedings. In this connection, we suggest to explicitly define the legal conditions and standards for Indigenous peoples’ participation, whether as amici curiae or as NDPs, so as to restrain arbitrators’ discretion and to inject more transparency into the ISDS decision-making process.

International investment law and international human rights law are traditionally parallel, and they have different emphasis and objectives. As Lorenzo Cotula observed, “[B]eyond diversity within human rights law, and within investment law, there are also questions about the relationship between the two” [27]. Concerning Indigenous peoples, according to the OECD, key international human rights instruments, such as the Indigenous and Tribal Peoples Convention and the UNDRIP, were rarely referred to by arbitrators in ISDS proceedings [28,29]. The reason is largely because human rights laws are usually not regarded as the ‘applicable laws’ to be invoked by investor-state tribunals. Such an artificial distinction is subject to challenge in terms of its legitimacy [30]. We advocate that, as a principle, international human rights rules shall be required to be substantially incorporated into the ISDS system. This stands for the direction for future ISDS reform aiming at significantly strengthening protection of Indigenous peoples affected by international investments. Counterclaims, allowing host states to sue foreign investors who fail to fulfill human rights obligations, must be supplemented as an important alternative to amicus curiae submissions in the ISDS system [29].

Moreover, the imposition of sanctions is an essential pillar of a systematic approach to human rights in international investment law and arbitration [30]. Most ISDS cases do not result in compensation by states or foreign investors to affected Indigenous peoples. Burlington Resources Inc. v Ecuador/Kichwa Indigenous People of Sarayaku v Ecuador is exceptional and noteworthy, where, because an investment project adversely affected the territory of Kichwa, the foreign investor took Ecuador to ISDS tribunal, and concurrently Ecuador was sued by Kichwa Indigenous people of Sarayaku in the Inter-American Court of Human Rights. The court later decided that Ecuador’s conduct amounted to a violation of the rights to consultation, to Indigenous communal property, and to cultural identity under relevant international law such as Article 21 of the American Convention on Human Rights, and therefore ordered Ecuador to pay $1.4 million compensation for damages, and to consult with the Sarayaku people on the removal of remaining explosives on the territory of the Sarayaku as well as on any type of investment with potential repercussions for their territory or affect essential aspects of their life [31]. This case indicates an alternative approach to seeking legal remedies: recourse to another dispute resolution mechanism to supplement the ISDS mechanism. We suggest that, when considering future ISDS reform, the ISDS mechanism is strengthened by resorting to other adjudicating institutions, such as regional human rights courts.

#### 4.2.2. Upgrading Substantive Rules in IIAs

At its 34th Session held in November 2017, Working Group III of UNCITRAL noted that “critical questions on possible ISDS reform involved underlying substantive rules”, and it “raised complex issues of public international law” such as that the reform should be government-led [32]. As aforementioned, when foreign investors initiate ISDS proceedings against host states, it is not just because the host states are to protect Indigenous peoples’ environment, mostly it is the result of host states’ failure to consult with and even carry out a process of FPIC prior to granting licences. For host states, the risk of facing compensation claims of foreign investors, or the domestic pressure caused by environmental damage and protests of Indigenous communities, can be very high so as to let any local governments take the possible “triple losing” scenario seriously. In view of the host states’ principal position in protecting Indigenous peoples and the environment, we believe upgrading substantive rules in IIAs is equally important to address existing problems of the ISDS system.

In particular, we suggest the incorporation of provisions dealing with land expropriation, natural resources exploration, compensation for environmental pollution and damages into the IIAs, especially BITs. Special treatments of Indigenous peoples’ rights will immune them from challenges by the non-discrimination principle or other provisions under the IIAs. As a matter of fact, a small number of modern IIAs, such as the Canada-Senegal BIT and the Canada-Peru FTA, adopt this approach. When negotiating the new United States-Mexico-Canada Agreement (USMCA), Canada proposed to set up a special chapter on protecting Indigenous rights. Though in the end the USMCA emerged without such a chapter, its ideals were “woven throughout” the fabric of the final deal [33]. For the first time, for instance, the preamble to the USMCA recognizes “the importance of increased engagement by Indigenous peoples in trade and investment…” At Canada’s insistence, USMCA also includes a general exception—applicable to the entire USMCA—for obligations to Indigenous peoples [34,35]. Additionally, in its Chapter 24 on Environment, there are several other special mentions of Indigenous rights. These new developments on substantive rules in IIAs would undoubtably help address institutional problems in the current weak protection of Indigenous peoples, and should therefore be encouraged.

#### 4.2.3. Emphasizing FPIC

Free, prior and informed Consent (FPIC) is one of the key principles that can ensure Indigenous peoples’ right to participation [35]. As an emerging standard in the dialogue on Indigenous peoples’ rights, it implies “genuine inclusion, disclosure, and respect for Indigenous peoples decision-making processes” [36]. It has been established not only in international agreements, but in soft law, notably the UNDRIP which requires that FPIC be obtained in matters of fundamental importance for their rights and well-being. The FAO further defines that “[F]or an FPIC process to be effective…, the way in which the process is conducted is paramount. The time allocated for the discussions among the Indigenous peoples, the cultural appropriateness of the way the information is conveyed, and the involvement of the whole community…, are all essential” [35]. In some ISDS proceedings, such as the Grand River Enterprises Six Nations v. United States of America case, the ICSID tribunal recognized the existence of customary international law norms concerning Indigenous peoples, including “the right to be consulted with respect to any project that may affect them” [36,37]. However, implementation of FPIC in past international investment development activities was neither easy nor found satisfactory by the Indigenous peoples.

We suggest the strengthening of future regulatory support of FPIC from three perspectives: (i) domestic law of host states. The tribunal in the Bear Creek case highlighted that the host state was “responsible for informing its citizens of State decisions, acts of public administration, and their effects” [38]. As host states are always in the dominant position, protecting Indigenous peoples’ rights to FPIC through their domestic laws would provide an important starting point [39]. In particular, the host-state government role in implementing FPIC prior to issuing licences to foreign investors must be highlighted. (ii) international investment law. Only a small number of existing IIAs require that Indigenous groups must be consulted before their regions are developed by foreign investments, and these provisions are usually vague and not mandatory. We suggest the incorporation of more explicit FPIC provisions, preferably as compulsory obligations, into investment treaties so as to ensure FPIC’s implementation at the transnational level. (iii) investor-state contracts. Investor-state contracts also interact with obligations under IIAs in complex ways, but they are concluded directly between host states and foreign investors without involving Indigenous communities in negotiations [39]. Hence, we suggest greater involvement of all stakeholders and higher transparency in the conclusion of investment contracts, especially in those contracts involving development of land and natural resources of Indigenous peoples. It is also desirable to conclude a trilateral agreement of development projects between foreign investors, government bodies, and authorized representatives of the Indigenous communities [40].

#### 4.2.4. Strengthening Foreign Investors’ Social Responsibilities

Though ensuring Indigenous peoples’ FPIC is mainly an obligation of the host states, the dissenting arbitrator in the Bear Creek case held that “foreign investors also play an important part in process of obtaining local trust” and found the ILO Convention 169 had legal effects on foreign investors as well [41]. We agree with this observation and, as there are an increasing number of companies that have already incorporated FPIC language into their policies and internal implementation guidelines [42], we suggest that FPIC shall be taken as part of foreign investors’ corporate social responsibilities (CSR).

As aforementioned, for-profit foreign investors naturally lack impetus to protect Indigenous peoples’ environment, therefore underlining their CSR in a legitimate way becomes desirable. We propose two major directions of future reform for this purpose: (i) incorporating CSR into IIAs. In its 2016 World Investment Report the UNCTAD examined ten recent BITs, seven of which were found containing CSR clauses that promote responsible investments [42]. The USMCA also highlighted CSR and Responsible Business Conduct in its Article 24.13. These latest developments indicate the future trend in rule-making. Because of the nature of IIAs, we believe that if most IIAs could incorporate such CSR obligations onto foreign investors, fewer disputes will occur over Indigenous peoples and their environment. (ii) advocating soft norms. Different from international investment law, the existing body of international environmental law partially “emerged on the basis of soft norms” [43] such as declarations and resolutions of the United Nations. Current regulations concerning CSR are also mainly soft norms other than hard laws. Thus, we suggest promoting obligatory awareness of key normative instruments of all stakeholders involved in the protection of Indigenous rights. The Guiding Principles for Business and Human Rights, the first corporate human rights responsibility initiative endorsed by the UN, for instance, are already respected by not only foreign investors, but also host states and home states of international investments. However, given that an instrument such as this is not legally binding, it would be desirable to enact more enforceable CSR regulations.

## 5. Conclusions

The unprecedented COVID-19 pandemic has impacted our world significantly. As the global pandemic continues wreaking havoc on the economy, governments of host states, especially those of developing and least developed countries along China’s Belt and Road Initiative, will be more eager to attract foreign capital other than to spend on environment protection. Indigenous peoples in these host states are therefore facing higher risks of being adversely affected by foreign investment activities. Past cases indicate that, as revealed and analyzed by the present research, the existing legal framework of international investment, including its ISDS mechanism, can hardly provide full and effective protection to affected Indigenous communities. Future reforms are necessary not only over technical matters such as facilitating Indigenous communities’ participation in ISDS proceedings and enhancing human rights weighting by ISDS tribunals, but also on the overall design and coordination of various IIAs, international environmental rules, as well as relevant legal instruments at the domestic and national levels. To conclude, for Indigenous peoples’ wellbeing and environment protection, a more sustainable international investment regime containing an effective ISDS mechanism is much desired by the international community.

## Figures and Tables

**Table 1:** Typical ISDS Cases Involving Indigenous Peoples between 2000 and 2020.

Claimant	Respondent	Decision Date	Arbitrating Authority	Treaty Basis	Indigenous Peoples Involved
Glamis Gold Ltd.	United States of America	June 2009	ICSID	NAFTA	Quechan Indian Tribe and other Native American Tribes
Grand River Enterprises Six Nations, Ltd., et al.	United States of America	January 2011	ICSID	NAFTA	Iroquois Confederacy (Haudenosaunee)
Bernhard von Pezold and others	Zimbabwe	July 2015	ICSID	Germany-Zimbabwe BIT and Switzerland-Zimbabwe BIT	The Chikukwa, Ngorima, Chinyai and Nyaruwa peoples
Crystallex International Corporation	Bolivarian Republic of Venezuela	April 2016	ICSID	Canada-Venezuela BIT	Indigenous population live in Imataca Forest Reserve
Pac Rim Cayman LLC	Republic of EI Salvador	October 2016	ICSID	CAFTA	Local inhabitants in Cabanas
Burlington Resources Inc.	Republic of Ecuador	August 2017	ICSID	Ecuador-United States BIT	Indigenous in Ecuadorian Amazon Region
Bear Creek Mining Corporation	Republic of Peru	November 2017	ICSID	Canada-Peru FTA	The Aymara population
Chevron Corporation and Texaco Petroleum Company	Republic of Ecuador	August 2018	PCA	Ecuador-United States BIT	Amazonian peoples, especially Indigenous peoples in Oriente area of Ecuador
Álvarez y Marín Corporación S.A. and others	Republic of Panama	September 2018	ICSID	Netherlands—Panama BIT and Central America-Panama FTA	Indigenous reserve known as the Comarca Ngäbe-Buglé
South American Silver Limited	The Plurinational State of Bolivia	November 2018	PCA	Bolivia-United Kingdom BIT	Aymara and Quechua

**Table 2:** Participation of Indigenous Peoples in ISDS Proceedings.

ISDS Cases	Participation of Indigenous Peoples
Glamis Gold, Ltd. v. United States of America	Friends of the Earth, Quechan Indian Nation, Parties Sierra Club and Earthworks, and National Mining Association submitted applications. Claimant objected to application of the Friends of the Earth, while Respondent fully supported amicus participation. Tribunal decided to accept and then considered each submission.
Grand River Enterprises Six Nations, Ltd., Et Al. v. United States of America	National Chief of the Assembly of First Nations made submission to the ISDS tribunal.
Bernhard Von Pezold and Others v. Zimbabwe	ECCHR and four Indigenous communities (Chikukwa, Ngorima, Chinyai and Nyaruwa peoples) applied to be amicus curiae and submitted several petitions. Tribunal denied the petition in its entirety.
Crystallex International Corporation v. Bolivarian Republic of Venezuela	No Indigenous communities applied for amicus curiae or NDP.
Pac Rim Cayman LLC v. Republic of El Salvador	No amicus curiae submissions were received. The Center for International Environmental Law (CIEL) applied as NDP. Tribunal admitted CIEL’s application as NDP, but decided it unnecessary to specifically consider CIEL’s case.
Burlington Resources Inc. v. Republic of Ecuador	No Indigenous communities applied for amicus curiae or NDP.
Bear Creek Mining Corp. v. Republic of Peru	Dhuma of Puno, Dr. Carlos Lopez and Columbia Center on Sustainable Investment (CCSI) submitted amicus curiae applications. Tribunal denied the application of CCSI.
Chevron Corporation and Texaco Petroleum Company v. The Republic of Ecuador	EarthRights International, Fundación Pachamama and The International Institute for Sustainable Development submitted petitions. Tribunal decided not to permit the participation of the Petitioners as amici curiae.
Álvarez y Marín Corporación and others v. Republic of Panama	No Indigenous communities applied for amicus curiae or NDP.
South American Silver Limited (Bermuda) v. The Plurinational State of Bolivia	No Indigenous communities applied for amicus curiae or NDP.

**Table 3:** Arbitral Results.

ISDS Cases	Arbitral Results
Glamis Gold, Ltd. v. United States of America	Decided in favor of state, dismissed Glamis’ claim almost in its entirety.
Grand River Enterprises Six Nations, Ltd., Et Al. v. United States of America	Decided in favor of state, but Tribunal stated “it would have been appropriate for governmental authorities in the United States to give greater recognition to, through appropriate consultations, the interests and concerns of Native American communities and entrepreneurs potentially affected by the MSA and related measures.”
Bernhard Von Pezold and others v. Zimbabwe	Decided in favor of investor, and ordered Zimbabwe “Restitution”.
Crystallex International Corporation v. Bolivarian Republic of Venezuela	Decided in favor of investor.
Pac Rim Cayman LLC v. Republic of El Salvador	Decided in favor of State.
Burlington Resources Inc. v. Republic of Ecuador	Decided in favor of investor.
Bear Creek Mining Corp. v. Republic of Peru	Decided in favor of investor, and specially mentioned “indigenous communities, irrespective whether they were in favor of or against the Project, are not respondent party in this arbitration.”
Chevron Corporation and Texaco Petroleum Company v. The Republic of Ecuador	Decided in favor of investor.
Álvarez y Marín Corporación and others v. Republic of Panama	Decided no jurisdiction.
South American Silver Limited (Bermuda) v. The Plurinational State of Bolivia	Decided in favor of investor.
